# Supplementary material for: Context-dependent choice and evaluation in real-world consumer behavior
Source: Sci Rep. 2022 Oct 22;12:17744. doi: 10.1038/s41598-022-22416-5 (PMC9588046; doi:10.1038/s41598-022-22416-5)
Supplement: Supplementary file 1 — Supplementary Information 1. [file 41598_2022_22416_MOESM1_ESM.pdf]

Supplementary Information

Follow-up Analyses of Yelp Choice Data

To address the possibility that the apparent difficulty in making value-maximizing choices in choice sets with higher average option ratings (e.g. Figure 2B) stems from confusion in discriminating the best option, we repeated the analysis of choice as a function of mean choice set rating, only considering choice sets where the second-best option is at least 1 star away from the highest-rated option (24% of choice sets; for example, a set with a highest-rated option of 4.5 stars where the mean rating of the next-best option is 3.5 or less). We found that the contextual effect of mean choice set rating upon ratings-maximizing "target" option selection rates was even stronger in this restricted dataset (β= -0.9001, *SE*=0.065, p<0.0001) suggesting against the possibility that these context effects arise from 'crowding' of options at the higher end of the rating scale. Further, to address the possibility that high-rated targets exert undue influence on our computed mean choice set rating, we tested if target choices could be predicted as a function of the mean choice set rating without the highest-rated (target) option, finding that the same context effects take hold (β= -0.284, *SE*=0.0346, p<0.0001).

In a follow-up analysis considering US cities, we used postal codes in conjunction with restaurant categories (as done with spatial clusters) to define choice sets (Figure S2A), replicating 1) the negative effect of mean choice set rating upon target choice proportion (Figure S2A; β= -0.3125, *SE*=0.0218, p<0.0001) and 2) the negative effect of set size upon target choice set on target choice proportion (Figure S2A; β= -0.7666, *SE*=0.0168, p<0.0001). However, the spatial size of—and accordingly, number of restaurants contained therein—of postal codes varies substantially across cities, resulting in uneven set size distributions across cities, and unrealistically large choice set sizes in some cities (Figure S1). Nonetheless, the robustness of these context effects to our geographic specification of choice set highlights the generalizability of our approach to understanding choice context effects.

Finally, to control for the preponderance of users who leave multiple ratings for the same restaurant (35% of users in our dataset), we have the same analysis of ratings-based choices, separately examining users who have at least one repetition of a restaurant in their ratings (35% of users in question, Figure S3A) and users who have no repetitions (the remaining 65% of users, Figure S3B). We found that the context effect of interest—fewer ratings-maximizing choices as the average value of the choice set increases—occurs in both subsets of the data, and these effects are statistically significant (and comparable) in both the "repeated choice" (β= -0.246, 95% CI:-0.3261, -0.1694, p<0.0001) and "non-repeated choice" (β= -0.2103, 95% CI:-0.3061, -0.1179, p<0.0001) subsets of the data.

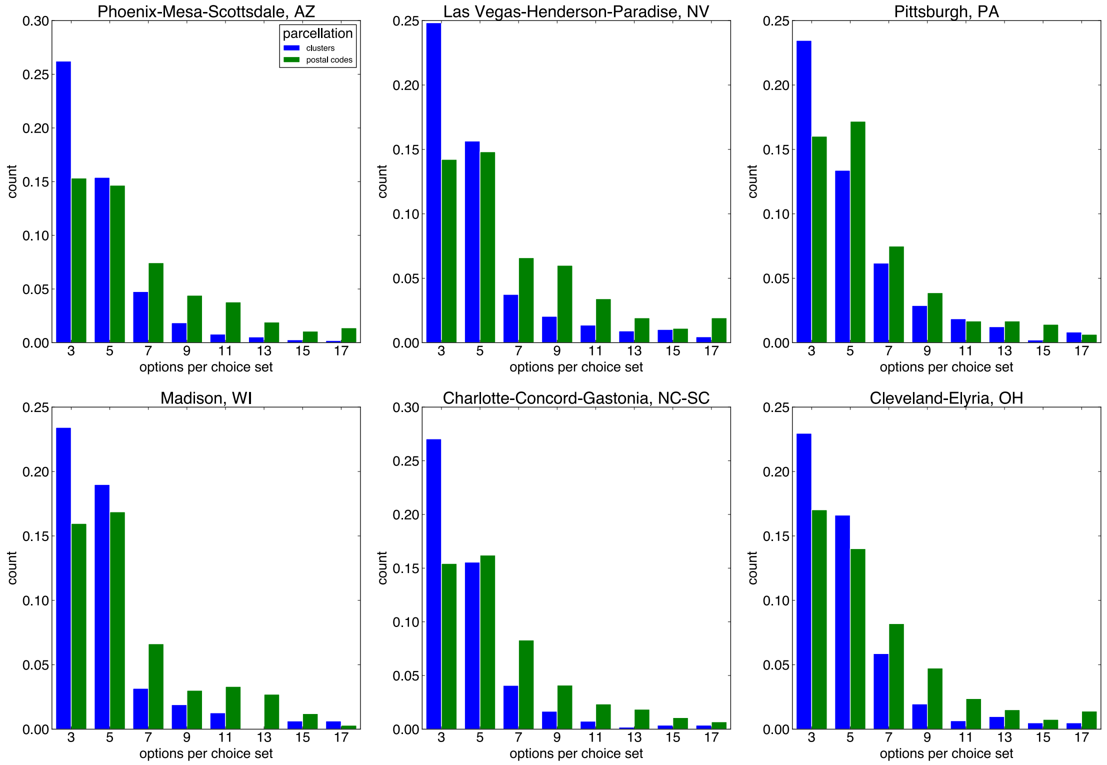

Figure S1. Distribution of the number of options per choice (defined by a spatial cluster and a restaurant category) set resulting from density-based clustering, plotted separately for each MSA.

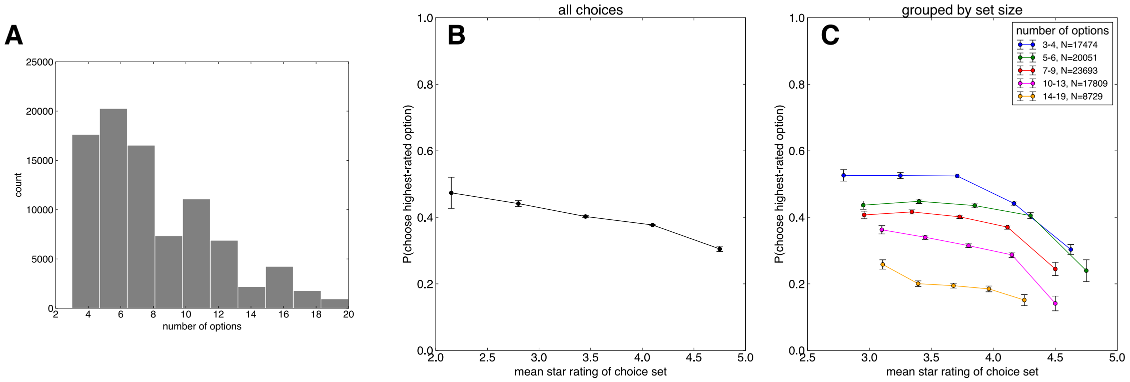

Figure S2. Analysis of choice sets resulting from postal code-based definition of choice sets. (A) Distribution of number of options per choice set, plotted for all MSAs. (B) Proportion of ratings-maximizing choices as a function of mean star rating of choice set for all choice sets. (C) Proportion of ratings-maximizing choices as a function of mean star rating, grouped by choice set size.

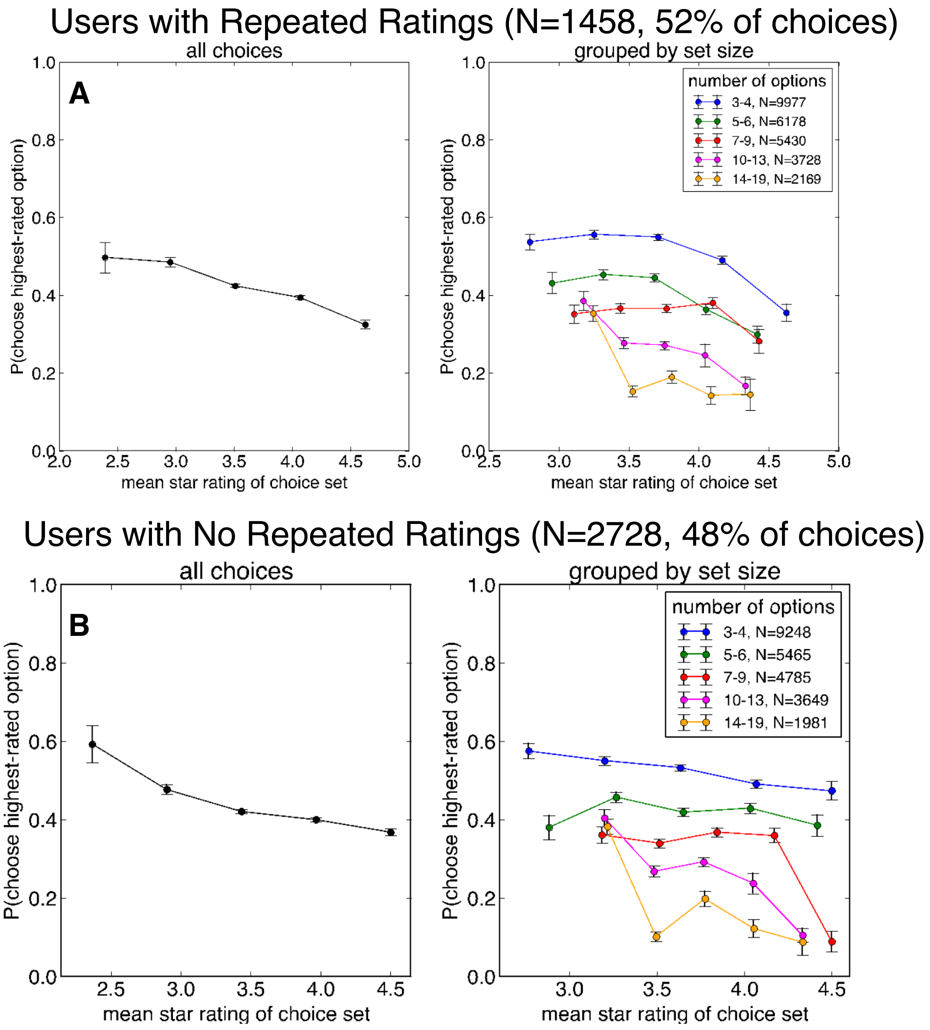

Figure S3. Proportion of ratings-maximizing choices as a function of mean star rating in the Yelp dataset, separately considering (A) Users with Repeated Ratings and (B) Users with No Repeated Ratings.

Table S1. Average number of restaurants per spatial cluster, and average number of restaurants per category per cluster (i.e., choice set size) resulting from the calculation of restaurant categories and neighborhoods.

	**Restaurants per Cluster**		**Restaurants per Category per Cluster**	
**MSA**	*Mean*	*SD*	*Mean*	*SD*
Charlotte-Concord-Gastonia, NC-SC	9.898	14.193	4.034	0.854
Phoenix-Mesa-Scottsdale, AZ	12.157	21.329	4.381	1.021
Cleveland-Elyria, OH	10.830	18.306	4.332	1.259
Las Vegas-Henderson-Paradise, NV	11.264	22.262	5.023	1.792
Pittsburgh, PA	12.264	27.147	4.871	1.418
Madison, WI	9.711	20.107	4.961	1.910
Toronto, ON	13.822	87.024	10.737	3.972
Calgary, AB	11.167	39.809	7.877	4.784

Table S2. Coefficient estimates for mixed-effects logistic regression predicting ratings-maximizing choices as a function of the difference between best-rated option and second-best-rated option and log-transformed choice set size.

Coefficient	*Estimate*	*95% CI*	*p-value*
(Intercept)	1.0338	0.8931, 1.1599	<0.0001*
difference between the top two highest-rated options	0.5182	0.4145, 0.6149	<0.0001*
number of options (log)	-0.9957	-1.0648, -0.908	<0.0001*

Table S3. Coefficient estimates for mixed-effects logistic regression predicting ratings-maximizing choices as a function of mean star rating of choice set (context effect) and log-transformed choice set size, controlling for variance in star ratings of the choice set as well as the price of the highest-rating option, and the log-transformed number of reviews of the highest-rated option in the Yelp ratings-based choice dataset.

Coefficient	*Estimate*	*95% CI*	*p-value*
(Intercept)	-0.4097	-0.6866, -0.1586	0.001*
difference between the top two highest-rated options	0.8849	0.7809, 0.9897	<0.0001*
mean star rating of choice set	-0.1175	-0.186, -0.0611	<0.0001*
number of options (log)	-0.9018	-0.9586, -0.8458	<0.0001*
number of reviews for highest-rated option	0.3629	0.342, 0.3818	<0.0001*
price of highest-rated option	0.0934	0.0594, 0.1246	<0.0001*

Table S4. Logistic regression predicting ratings-maximizing Check-in choices as a function of mean star rating of choice set (context effect) and log-transformed choice set size, the price of the highest-rating option, and the log-transformed number of reviews of the highest-rated option in the Yelp Checkins-based choice dataset.

Coefficient	*Estimate*	*95% CI*	*p-value*
(Intercept)	0.119188	0.111767, 0.126623	<0.001
mean star rating of choice set	-0.017767	-0.025576, -0.00987	<0.001
number of options (log)	-0.063328	-0.065914, -0.055814	<0.001
number of reviews for highest-rated option	0.053449	0.050901, 0.061123	<0.001
price of highest-rated option	0.00148	-0.006166, 0.009129	0.7
variance of star ratings of choice set	-0.001514	-0.009204, 0.006273	0.711

Table S5. Logistic regression predicting ratings-maximizing choices as a function of mean star rating of choice set (context effect) and log-transformed choice set size, the price of the highest-rating option, and the log-transformed number of reviews of the highest-rated option in the Deliveroo orders dataset.

Coefficient	*Estimate*	*95% CI*	*p-value*
(Intercept)	0.119188	0.111767, 0.126623	<0.001
mean star rating of choice set	-0.017767	-0.025576, -0.00987	<0.001
number of options (log)	-0.063328	-0.065914, -0.055814	<0.001
number of reviews for highest-rated option	0.053449	0.050901, 0.061123	<0.001
price of highest-rated option	0.00148	-0.006166, 0.009129	0.7
variance of star ratings of choice set	-0.001514	-0.009204, 0.006273	0.711

Table S6. Coefficient estimates for mixed-effects logistic regression predicting ratings-maximizing choices conditioned on users choosing one of the two highest-rated options in the set, as a function of the mean star rating of the distractor items (the sub-top-two-highest rated options), controlling for log-transformed choice set size, the price of the highest-rating option, and the log-transformed number of reviews of the highest-rated option in the Yelp ratings-based choice dataset.

Coefficient	*Estimate*	*95% CI*	*p-value*
(Intercept)	1.5484	1.2613, 1.8123	<0.0001*
mean of distractor options	-0.482	-0.5566, -0.4	<0.0001*
number of options (log)	-0.8569	-0.9589, -0.7197	<0.0001*
number of reviews for highest-rated option	0.3582	0.3071, 0.3907	<0.0001*
price of highest-rated option	0.1367	0.0975, 0.1779	<0.0001*

Table S7. Coefficient estimates for mixed-effects logistic regression predicting Check-in choices conditioned on users choosing one of the two highest-rated options in the set, as a function of the mean star rating of the distractor items (the sub-top-two-highest rated options), controlling for log-transformed choice set size, the price of the highest-rating option, and the log-transformed number of reviews of the highest-rated option in the Yelp Check-in-based choice dataset.

Coefficient	*Estimate*	*95% CI*	*p-value*
(Intercept)	0.9683	0.9109, 1.0269	<0.0001*
mean of distractor options	-0.497	-0.5131, -0.4802	<0.0001*
number of options (log)	-0.7764	-0.7956, -0.7547	<0.0001*
number of reviews for highest-rated option	0.4708	0.4613, 0.4792	<0.0001*
price of highest-rated option	0.1327	0.1233, 0.1425	<0.0001*

Table S8. Coefficient estimates for mixed-effects logistic regression predicting ratings-maximizing choices as a function of mean star rating of choice set (context effect) and log-transformed choice set size, the price of the highest-rating option, and the log-transformed number of reviews of the highest-rated in the Choice Experiment.

Coefficient	*Estimate*	*95% CI*	*p-value*
(Intercept)	3.8553	2.3001, 6.2506	<0.0001*
mean star rating of choice set	-0.8538	-1.321, -0.4892	<0.0001*
number of options (log)	-0.2311	-0.404, -0.0877	<0.0001*
number of reviews for highest-rated option	0.6213	0.4194, 0.9555	<0.0001*
price of highest-rated option	-0.1629	-0.3181, -0.0373	0.001*
variance of star ratings of choice set	0.0116	-0.2272, 0.2906	0.462
